# Contrast Extravasation on Computed Tomography Angiography as a Predictor of Hematoma Expansion and Outcomes in Spontaneous Intracerebral Hemorrhage: A Retrospective Study

**DOI:** 10.7759/cureus.95853

**Published:** 2025-10-31

**Authors:** Alhanouf H Hatim, Danah K Kabrah, Mohamed E Hassan, Hamad M Qabha

**Affiliations:** 1 Radiology, King Fahad General Hospital, Jeddah, SAU; 2 Radiology, King Abdulaziz Specialist Hospital, Taif, SAU; 3 Radiology, Prince Mohammed bin Abdulaziz National Guard Hospital, Medina, SAU; 4 Radiology, Dallah Hospital, Riyadh, SAU

**Keywords:** anticoagulant use, computed tomography angiography, contrast extravasation, hematoma expansion, intracerebral hemorrhage, middle east, outcomes, predictors, spot sign, systolic blood pressure

## Abstract

Background: Contrast extravasation (“spot sign”) on computed tomography angiography (CTA) is a key imaging biomarker of active bleeding in spontaneous intracerebral hemorrhage (ICH) and is strongly associated with hematoma expansion and poor outcomes. However, data from Middle Eastern populations remains limited. This study aimed to identify the clinical and radiologic predictors of contrast extravasation and to assess its association with hematoma expansion and functional outcomes among patients with spontaneous ICH.

Materials and methods: We conducted a multicenter retrospective cohort study of adult patients with spontaneous ICH who underwent non-contrast head CT and CTA at presentation between January 2020 and December 2024. Baseline demographic, clinical, and imaging data were collected. Contrast extravasation was defined as a focus of hyperattenuation ≥120 HU within the hematoma on CTA, discontinuous from normal vessels. The primary outcome was the presence of contrast extravasation. Secondary outcomes included hematoma expansion on follow-up CT and poor functional outcome at 90 days. Independent predictors were identified using multivariable logistic regression.

Results: A total of 450 patients (mean age 66.1 ± 12.0 years; 58.0% men) were included. Contrast extravasation was observed in 141 patients (31.3%). Independent predictors of contrast extravasation were larger baseline hematoma volume (adjusted odds ratio (aOR) 1.32 per 10-mL increase; 95% CI, 1.18-1.47; P < 0.001), higher systolic blood pressure (SBP) (aOR 1.19 per 10-mm Hg increase; 95% CI, 1.05-1.35; P = 0.006), and anticoagulant use (aOR 2.42; 95% CI, 1.50-3.90; P < 0.001). Contrast extravasation was strongly associated with hematoma expansion (aOR 2.94; 95% CI, 1.82-4.75; P < 0.001) and poor 90-day functional outcome (aOR 1.68; 95% CI, 1.06-2.66; P = 0.028). In-hospital mortality was higher among spot-positive patients (18.0% vs. 6.0%).

Conclusions: Contrast extravasation on CTA was present in nearly one-third of patients with spontaneous ICH in this Saudi multicenter cohort and independently predicted hematoma expansion and unfavorable outcomes. Larger hematoma volume, elevated SBP, and anticoagulant therapy were the strongest predictors. Routine assessment for the spot sign during acute ICH imaging may enhance early risk stratification and guide targeted management strategies.

## Introduction

Spontaneous intracerebral hemorrhage (ICH) remains one of the most devastating forms of stroke, accounting for approximately 10-15% of all strokes worldwide but responsible for a disproportionately high rate of mortality and disability [1,2]. Despite advances in neurocritical care and imaging, the early course of ICH is often characterized by progressive hematoma enlargement, which occurs in up to one-third of patients and is a significant determinant of neurological deterioration and poor outcomes [3,4]. Identifying patients at risk of hematoma expansion at presentation is therefore critical for optimizing management and improving prognosis.

Computed tomography angiography (CTA) has emerged as a rapid, widely available imaging modality for assessing ongoing bleeding in ICH. The “spot sign,” a focus of contrast extravasation within the hematoma on CTA, reflects active contrast leakage from fragile, ruptured vessels and has been consistently associated with early hematoma growth, neurological worsening, and poor functional outcomes [1-4].

Evidence from multicenter studies indicates that the presence of a spot sign is among the most powerful imaging predictors of hematoma expansion, with odds ratios (ORs) ranging from 3 to 5. Consequently, the spot sign has become an important biomarker in ICH research and a potential target for hemostatic and blood pressure-lowering interventions [4,5].

However, most existing evidence originates from North American, European, and East Asian populations, while data from Middle Eastern and Saudi cohorts remain scarce [4-9]. Regional differences in hypertension control, anticoagulant use, and imaging practices may influence both the prevalence and predictive value of the spot sign.

Understanding the predictors and prognostic significance of contrast extravasation in this regional population may help refine risk stratification, guide resource allocation, and inform the development of national ICH management pathways. Therefore, we conducted a multicenter retrospective study to identify the clinical and radiologic predictors of contrast extravasation on CTA in patients with spontaneous ICH and to examine its association with hematoma expansion and functional outcomes.

## Materials and methods

Study design and setting

This multicenter retrospective cohort study was conducted across hospitals within the Eastern Health Cluster, Kingdom of Saudi Arabia. It included all adult patients presenting with spontaneous ICH who underwent both non-contrast head CT and CTA at presentation. Data were obtained from radiology databases and electronic medical records between January 2020 and December 2024. The study protocol was approved by the Ethics Committee of the Ministry of Health, Kingdom of Saudi Arabia (approval number: REC-2023-18127), and informed consent was waived due to the study's retrospective nature.

Study population

Eligible patients were aged 18 years or older and had a diagnosis of primary spontaneous ICH confirmed on baseline non-contrast CT. Patients were excluded if the hemorrhage was secondary to trauma, tumor, vascular malformation, or hemorrhagic transformation of ischemic stroke. Those without baseline or follow-up CT imaging, or with poor-quality CTA scans that precluded evaluation for contrast extravasation, were also excluded. After applying these criteria, 450 consecutive patients were included in the final analysis.

Imaging acquisition and analysis

All patients underwent non-contrast CT followed by CTA of the head using multidetector CT scanners available at participating hospitals within the Eastern Health Cluster. Standardized imaging protocols were implemented across sites, with a slice thickness of 0.625-1.0 mm and administration of 50-70 mL of non-ionic iodinated contrast at a rate of 4-5 mL/s, followed by a 40-mL saline flush. Two experienced neuroradiologists, each with at least five years of experience, independently reviewed the CT and CTA images while blinded to clinical and outcome data. Any discrepancies were resolved by consensus.

The spot sign was defined as one or more foci of contrast pooling within the hematoma that were located within the contour of the hemorrhage, discontinuous from normal or abnormal vessels, and showing attenuation ≥120 Hounsfield units (HU) on CTA. Hematoma volume was measured on baseline and follow-up CT using the ABC/2 method. Hematoma expansion was defined as either an absolute increase of more than 6 mL or a relative increase of more than 33% in hematoma volume on follow-up CT obtained within 24 ± 12 hours.

Clinical and radiologic variables

Demographic, clinical, and laboratory data were extracted from electronic health records, including age, sex, baseline Glasgow Coma Scale (GCS) score, and systolic blood pressure (SBP). Comorbidities such as hypertension and diabetes, as well as the use of antiplatelet or anticoagulant therapy at presentation, were recorded. Imaging characteristics, including hematoma location, baseline volume, and intraventricular extension (IVH), were also documented. Functional outcome was assessed using the modified Rankin Scale (mRS) at 90 days, obtained during outpatient follow-up visits or structured telephone interviews. Poor functional outcome was defined as an mRS score of 4 or higher.

Outcome measures

The primary outcome was the presence of contrast extravasation (spot sign) on initial CTA. Secondary outcomes included hematoma expansion on follow-up imaging and poor functional outcome at 90 days, defined as an mRS score ≥4. In-hospital mortality was recorded as an exploratory outcome.

Statistical analysis

Continuous variables were summarized as mean ± standard deviation (SD) or median (interquartile range (IQR)), as appropriate, and categorical variables were presented as counts and percentages. Comparisons between patients with and without a spot sign were performed using Student’s t-test or the Mann-Whitney U test for continuous variables and the χ² test or Fisher’s exact test for categorical variables.

Multivariable logistic regression was used to identify independent predictors of contrast extravasation, including variables with a P-value <0.10 in univariate analysis or those considered clinically relevant (age, sex, GCS, SBP, baseline hematoma volume, anticoagulant use, and IVH). Separate logistic regression models were constructed to assess the association between the spot sign and hematoma expansion, as well as poor 90-day functional outcome, after adjustment for confounders. Results were reported as ORs with 95% confidence intervals (CIs). A two-tailed P < 0.05 was considered statistically significant. Statistical analyses were performed using SPSS Statistics version 29.0 (IBM Corp., Released 2022. IBM SPSS Statistics for Windows, Version 29.0. Armonk, NY: IBM Corp.) and R version 4.3.0 (R Foundation for Statistical Computing, Vienna, Austria).

## Results

Study population

A total of 450 patients with spontaneous ICH who underwent admission non-contrast CT and CTA were included in the analysis. The mean age was 66.1 ± 12.0 years, and 58.0% were men. Hypertension was present in 74.0% of patients, diabetes mellitus in 22.0%, and 22.0% were receiving oral anticoagulation at presentation. The mean initial GCS score was 13.0 ± 2.5, and the mean SBP at presentation was 169.7 ± 27.6 mm Hg. The mean baseline hematoma volume was 26.5 ± 17.3 mL, and IVH was observed in 28.0% of cases (Table 1). Overall, 141 patients (31.3%) demonstrated contrast extravasation, or “spot sign,” on CTA (Table 2).

**Table 1:** Baseline characteristics of the study population according to spot sign status Data are presented as mean ± SD or number (percentage) unless otherwise indicated. GCS: Glasgow Coma Scale, SBP: systolic blood pressure, SD: standard deviation, mL: milliliter, mm Hg: millimeters of mercury

Variable	Overall (N = 450)	Spot sign (+) (N = 141)	Spot sign (−) (N = 309)
Age, years	66.1 ± 12.0	65.2 ± 11.9	66.5 ± 12.1
Male sex	261-58.0%	85-60.3%	176-57.0%
Hypertension	333-74.0%	106-75.2%	227-73.5%
Diabetes	99-22.0%	29-20.6%	70-22.7%
Anticoagulant use (any)	99-22.0%	48-34.0%	51-16.5%
Antiplatelet use	149-33.1%	43-30.5%	106-34.3%
GCS score	13.0 ± 2.5	12.4 ± 2.6	13.2 ± 2.4
SBP, mm Hg	169.7 ± 27.6	174.9 ± 28.2	167.4 ± 27.2
Baseline hematoma volume, mL	26.5 ± 17.3	34.6 ± 19.5	22.9 ± 15.1
Intraventricular hemorrhage present	126-28.0%	49-34.8%	77-24.9%

**Table 2:** Imaging features and prevalence of the spot sign Data are presented as median (IQR) or number (percentage) unless otherwise indicated. IVH: intraventricular hemorrhage, IQR: interquartile range, CTA: computed tomography angiography, mL: milliliter

Characteristic	Overall
Spot sign prevalence	141-31.3%
Median baseline volume, mL (IQR)	23 (15-38)
IVH prevalence	126-28.0%
Multiphase CTA performed	312-69.3%

Comparison between patients with and without contrast extravasation

Patients with a spot sign differed significantly from those without (Table 3). They had larger baseline hematoma volumes (mean, 34.6 mL vs. 22.9 mL; P < 0.001), higher SBP at presentation (174.9 vs. 167.4 mm Hg; P = 0.014), and lower GCS scores (12.4 vs. 13.2; P = 0.008). Anticoagulant use was more common among spot-positive patients (34.0% vs. 16.5%; P < 0.001). The spot sign was also more frequently observed in cases with intraventricular hemorrhage (34.8% vs. 24.9%; P = 0.031). No significant differences were observed between groups regarding age, sex, or diabetes mellitus.

**Table 3:** Univariate comparison of clinical and imaging variables between patients with and without spot sign Data are presented as means unless otherwise indicated. GCS: Glasgow Coma Scale, SBP: systolic blood pressure, IVH: intraventricular hemorrhage, mL: milliliter

Variable	Overall	Spot (+)	Spot (−)	P-value
Age, years	66.1	65.2	66.5	0.231
Male sex %	58.0	60.3	57.0	0.537
GCS	13.0	12.4	13.2	0.008
SBP, mm Hg	169.7	174.9	167.4	0.014
Baseline volume, mL	26.5	34.6	22.9	<0.001
IVH %	28.0	34.8	24.9	0.031
Anticoagulant use %	22.0	34.0	16.5	<0.001

Predictors of contrast extravasation

In the multivariable logistic regression analysis (Table 4), baseline hematoma volume, SBP, and anticoagulant use remained independent predictors of contrast extravasation. Each 10-mL increase in baseline hematoma volume was associated with a 32% higher odds of having a spot sign (adjusted OR (aOR), 1.32; 95% CI, 1.18-1.47; P < 0.001). Each 10-mm Hg increase in SBP was associated with a 19% higher odds (aOR, 1.19; 95% CI, 1.05-1.35; P = 0.006). Anticoagulant therapy was independently associated with a more than twofold increase in risk (aOR, 2.42; 95% CI, 1.50-3.90; P < 0.001). Lower GCS scores were also modestly associated with spot sign presence (P = 0.031), whereas age, sex, and IVH were not significant predictors after adjustment.

**Table 4:** Multivariable logistic regression analysis for predictors of contrast extravasation (spot sign) aORs with 95% CIs are shown. aORs: adjusted odds ratio, CI: confidence interval, GCS: Glasgow Coma Scale, IVH: intraventricular hemorrhage, SBP: systolic blood pressure, mL: milliliter, y: years

Variable	aOR (95% CI)	P-value
Age (per 10 y)	0.93 (0.79-1.08)	0.337
Male sex	1.08 (0.72-1.64)	0.711
GCS (per point)	0.89 (0.80-0.99)	0.031
SBP (per 10 mm Hg)	1.19 (1.05-1.35)	0.006
Baseline volume (per 10 mL)	1.32 (1.18-1.47)	<0.001
Anticoagulant use	2.42 (1.50-3.90)	<0.001
IVH present	1.41 (0.91-2.19)	0.121

Association of contrast extravasation with hematoma expansion and outcomes

Contrast extravasation was strongly associated with both hematoma expansion and poor functional outcomes (Table 5). Hematoma expansion occurred significantly more often in patients with a spot sign, with an unadjusted OR of 3.68 (95% CI, 2.40-5.65; P < 0.001). After adjustment for age, baseline hematoma volume, and GCS, the association remained robust (aOR, 2.94; 95% CI, 1.82-4.75; P < 0.001). The presence of a spot sign was also linked to a worse 90-day functional outcome (mRS score ≥4), with an aOR of 1.68 (95% CI, 1.06-2.66; P = 0.028). In-hospital mortality was higher among patients with contrast extravasation compared with those without (18.0% vs. 6.0%), consistent with the observed associations with hematoma expansion and neurological deterioration.

**Table 5:** Association of spot sign with hematoma expansion and 90-day functional outcome ORs with 95% CIs are shown for unadjusted and adjusted analyses. Hematoma expansion is defined as an absolute increase of >6 mL or a relative increase of >33% on follow-up CT. CT: computed tomography, CI: confidence interval, mRS: modified Rankin Scale, ORs: odds ratio

Outcome	Unadjusted OR (95% CI)	Adjusted OR (95% CI)	P-value
Hematoma expansion	3.68 (2.40-5.65)	2.94 (1.82-4.75)	<0.001
90-day poor outcome (mRS 4–6)	2.11 (1.43-3.11)	1.68 (1.06-2.66)	0.028

## Discussion

In this multicenter retrospective cohort from Saudi Arabia, we found that contrast extravasation (spot sign) was present in approximately one-third of patients with spontaneous ICH. Independent predictors of spot sign included larger baseline hematoma volume, higher SBP, and anticoagulant therapy at presentation. Moreover, the presence of a spot sign was strongly associated with hematoma expansion and poor 90-day functional outcomes, even after adjustment for clinical severity and baseline hematoma size.

Our findings align with those of landmark studies that established the spot sign as a robust predictor of hematoma expansion and poor prognosis. In previous cohorts, the prevalence of spot sign ranged from 25% to 35%, comparable to the 31% observed in our study [8-12]. Similarly, the independent associations we identified, particularly with baseline hematoma volume and anticoagulant use, have been consistently reported across both Western and Asian populations [10-13].

The significant relationship between elevated SBP and contrast extravasation may reflect active bleeding dynamics and vascular fragility. This observation reinforces the clinical rationale for early and intensive blood pressure control. Although lower GCS scores were associated with spot sign, the relationship was weaker, likely reflecting the influence of hematoma location and secondary mass effect on neurological status.

From a radiologic perspective, our results underscore the value of CTA as an integral component of initial ICH imaging. CTA enables early identification of patients at high risk of hematoma expansion, providing an immediate, noninvasive indicator of ongoing bleeding that can inform both triage and therapeutic decisions.

In clinical practice, spot-positive patients could be prioritized for early hemostatic interventions, such as reversal of anticoagulation or administration of procoagulant agents, along with more aggressive blood pressure management and close hemodynamic monitoring [12,14]. Repeat imaging surveillance may also be warranted to detect hematoma progression. Given that CTA is readily available, incorporating standardized spot sign assessment into ICH management protocols could enhance early risk stratification and improve patient outcomes.

This study has several limitations that should be acknowledged. First, its retrospective design introduces the potential for selection and information bias. Second, the timing of follow-up CT scans was not fully standardized, which could influence estimates of hematoma expansion. Third, although multicenter, the cohort was derived from a single regional healthcare system, which may limit the generalizability of our findings to other populations or healthcare settings. Finally, although the ABC/2 method was uniformly applied for volume estimation, advanced volumetric software could provide more precise measurements.

Prospective validation through a national multicenter registry is warranted to confirm the predictive utility of the spot sign in the Saudi population. Integration of CTA findings with emerging imaging biomarkers, such as permeability imaging, radiomic texture analysis, and dual-energy CT parameters, may further enhance predictive accuracy. Additionally, future clinical trials in the region should evaluate whether targeted hemostatic therapy guided by spot sign status can improve functional outcomes.

## Conclusions

Contrast extravasation on CTA is a common and powerful imaging marker of poor prognosis in spontaneous ICH. In this cohort, larger hematoma volume, higher SBP, and anticoagulant use independently predicted the presence of a spot sign. Patients with a spot sign were nearly three times more likely to experience hematoma expansion and twice as likely to have poor 90-day outcomes. Routine assessment for spot sign within emergency imaging workflows may enhance early prognostication and support individualized management strategies for patients with ICH in Saudi Arabia. Based on our findings, we propose that the identification of a spot sign on initial CTA should trigger a structured management protocol. This could include expedited reversal of anticoagulation, more aggressive blood pressure control targeting specific systolic thresholds, and scheduling an early follow-up CT scan to monitor for expansion, thereby enabling timely intervention.
